# Stroma-derived extracellular vesicles deliver tumor-suppressive miRNAs to pancreatic cancer cells

**DOI:** 10.18632/oncotarget.23532

**Published:** 2017-12-20

**Authors:** Song Han, David H. Gonzalo, Michael Feely, Carlos Rinaldi, Sayali Belsare, Haiyan Zhai, Krishan Kalra, Michael H. Gerber, Christopher E. Forsmark, Steven J. Hughes

**Affiliations:** ^1^ Department of Surgery, University of Florida College of Medicine, Gainesville, FL, USA; ^2^ Department of Pathology, University of Florida College of Medicine, Gainesville, FL, USA; ^3^ Department of Biomedical Engineering, University of Florida College of Medicine, Gainesville, FL, USA; ^4^ BioGenex Laboratories, Fremont, CA, USA; ^5^ Division of Gastroenterology, University of Florida College of Medicine, Gainesville, FL, USA

**Keywords:** pancreatic cancer, microenvironment, exosomes, microvesicles, miR-145

## Abstract

The biology of tumor-associated stroma (TAS) in pancreatic ductal adenocarcinoma (PDAC) is not well understood. The paradoxical observation that stroma-depletion strategies lead to progression of PDAC reinforced the need to critically evaluate the functional contribution of TAS in the initiation and progression of PDAC. PDAC and TAS cells are unique in their expression of specific miRNAs, and this specific miRNA expression pattern alters host to tumor microenvironment interactions. Using primary human pancreatic TAS cells and primary xenograft PDAC cells co-culture, we provide evidence of miRNA trafficking and exchanging between TAS and PDAC cells, in a two-way, cell-contact independent fashion, via extracellular vesicles (EVs) transportation. Selective packaging of miRNAs into EVs led to enrichment of stromal specific miR-145 in EVs secreted by TAS cells. Exosomes, but not microvesicles, derived from human TAS cells demonstrated a tumor suppressive role by inducing PDAC cell apoptosis. This effect was mitigated by anti-miR-145 sequences. Our data suggest that TAS-derived miRNAs are delivered to adjacent PDAC cells via exosomes and suppress tumor cell growth. These data highlight that TAS cells secrete exosomes carrying tumor suppressive genetic materials, a possible anti-tumor capacity. Future work of the development of patient-derived exosomes could have therapeutic implications for unresectable PDAC.

## INTRODUCTION

By volume, the majority of pancreatic adenocarcinoma (PDAC) is tumor-associated stroma (TAS). This extensive desmoplasia is driven by the activation and proliferation of pancreatic stellate cells (PSCs) that assume a myofibroblast phenotype and contribute to the production of a dense extracellular matrix [1]. For the past several years, a large body of evidence suggested that TAS contributes to the PDAC phenotype of chemoresistance, invasion, metastasis, and immune tolerance [2]. For example, a stroma-modulation strategy via hedgehog pathway inhibition improved chemotherapy delivery and survival in a murine model [3]. But recently, multiple groups challenged this paradigm [4–6]; they demonstrated that depletion of PDAC stroma or disruption of hedgehog signaling in differing *in vivo* models resulted in acceleration of tumor progression. These studies provide compelling evidence of the importance, complexity, and plasticity of TAS, that reinforces the need for improving our understanding of interactions between TAS and PDAC cells with translational implications for future therapy [7].

Germane to this concept and the present study, a recently identified mechanism of cellular communication is the exchange of microRNAs (miRNAs) between cells. We previously demonstrated distinct epithelial and stromal miRNA expression patterns in pancreatic cancer both in *in vitro* cultured cells and in human specimens of PDAC. Specifically, miR-205 and miR-200 family members (in particular miR-200b and miR-200c) were exclusively expressed by pancreatic cancer epithelial cells, and miR-145 and miR-199 family members (miR-199a and miR-199b) were exclusively expressed by TAS cells [8]. Our monolayer co-culture data suggested that an exchange of these miRNAs could be occurring between these cell types within the PDAC microenvironment, however, an alternative mechanism such as other paracrine signals that influenced expression could not be excluded.

The membrane-bound extracellular vesicles (EVs) collectively represent particles of differing mechanistic origin and include both microvesicles (MVs) and exosomes (EXOs) are now being recognized as potential mechanisms for the shuttling of molecules including DNA, RNA, protein, and microRNA between cells [9, 10]. This role of EVs as a mechanism of intercellular communication between tumor cells and the local microenvironment and distant organs has become the subject of intense interest in recent studies [11, 12]. Exosomes contain transmembrane and membrane-anchored proteins, and are proven to enhance endocytosis, thus promoting the delivery of their internal content [13]. Recent work using exosomes derived from normal fibroblasts engineered with shRNA specific to oncogenic Kras suppressed cancer in mouse models of pancreatic cancer and significantly increased overall survival [14]. Here, we aimed to confirm that the exchange of miRNAs between TAS cells and PDAC cells is mediated by EVs, and to further understand how such an exchange might impact the biology of PDAC. These results have important implications for the development of exosome-based therapeutic strategies.

## RESULTS

### A miRNA exchange occurs between cell types in an *in vitro* model of the tumor microenvironment

**Figure 1:**
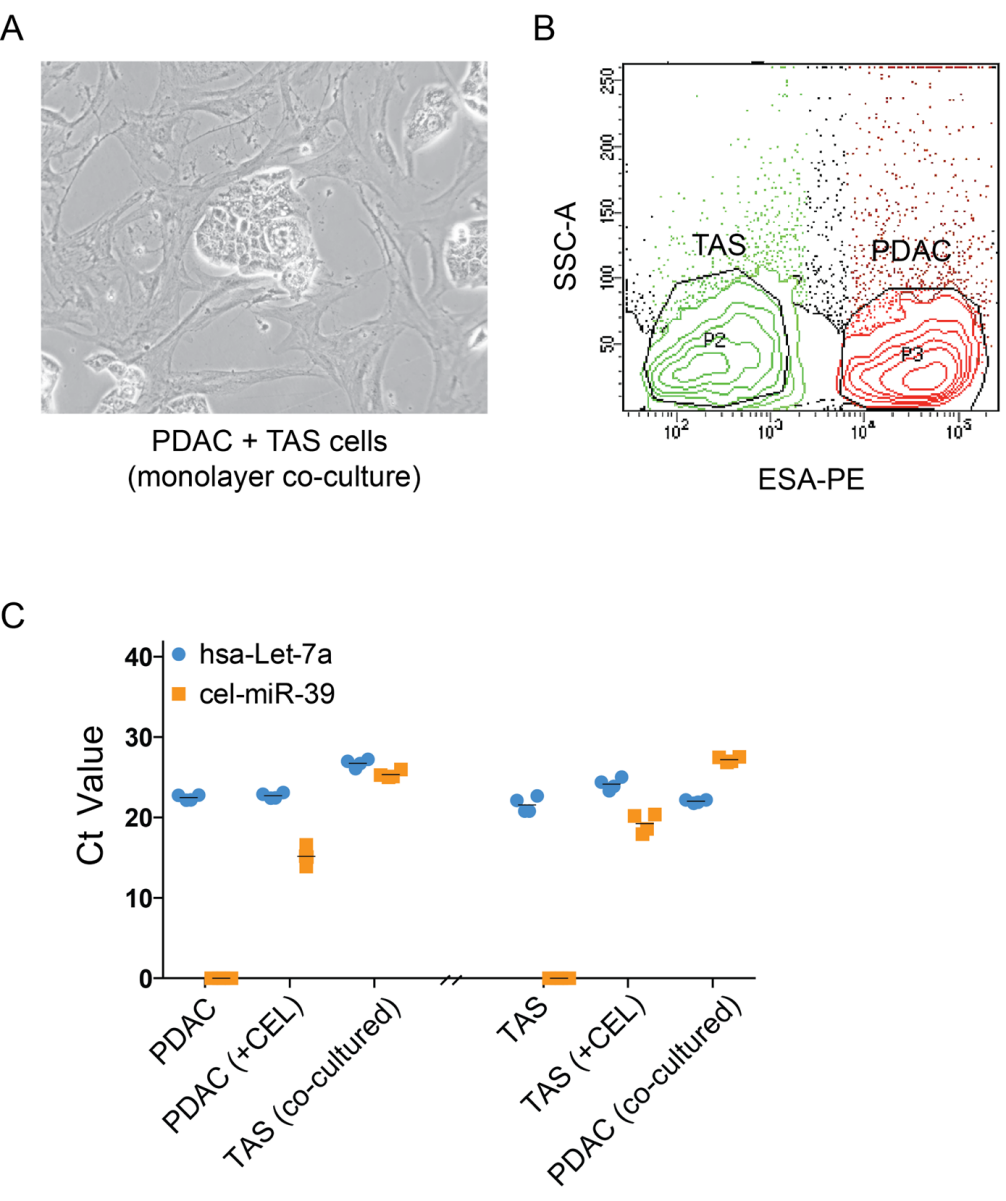
Exchanges of miRNAs, rather than changes in intrinsic expression, occur between PDAC and TAS cells (**A**) Phase contrast imaging of monolayer, co-cultures of PDAC and TAS cells demonstrates clusters of cancer cells (PDAC) surrounded by TAS cells. (**B**) Flow cytometric cell sorting for the separation of PDAC (ESA+) cells and TAS (ESA–) cells after monolayer co-culture. (**C**) qPCR determination of cel-miR-39-3p expression in transfected (+CEL) and monolayer co-cultured cells. hsa-let-7a-5p, a miRNA abundantly and consistently expressed in both types of cells serves as an internal control. Each plotted point represents the Ct value of duplicate PCR reactions from two individual transfection and monolayer co-culture experiments. +CEL: cel-miR-39-3p transfected cells.

We previously identified the presence of TAS-specific miRNAs, such as miR-145, in PDAC cells following *in vitro* co-culture, and vice versa [8]. To confirm that this finding is due to an exchange of miRNA between the two types of cells and not due to changes in expression in one cell type in response to other signals (i.e secreted proteins), a template of non-human miRNA mimic from *C. elegans*, Cel-miR-39-3p (CEL), was expressed in donor cells (i.e. TAS cells) via transfection prior to co-culture with recipient cells (i.e. PDAC cells) as shown in Figure 1A. The two cell types were then effectively separated as previously described [8] by FACScan-based cell sorting using ESA reactivity (Figure 1B). qPCR detected cel-miR-39-3p expression in both donor (CEL-transfected) and monolayer co-cultured recipient cells (Figure 1C). This experiment was carried out both ways, using either CEL-transfected PDAC or CEL-transfected TAS cells as the donor cells. These data confirm a reciprocal exchange of miRNA occurs between PDAC and TAS cells within the monolayer co-culture milieu. Thus, the increase in miRNA expression levels in counterpart cells following co-culture as we previously observed is not simply the result of protein-based or other signals driving changes in gene expression.

### miRNA exchange is cell-cell contact independent

**Figure 2:**
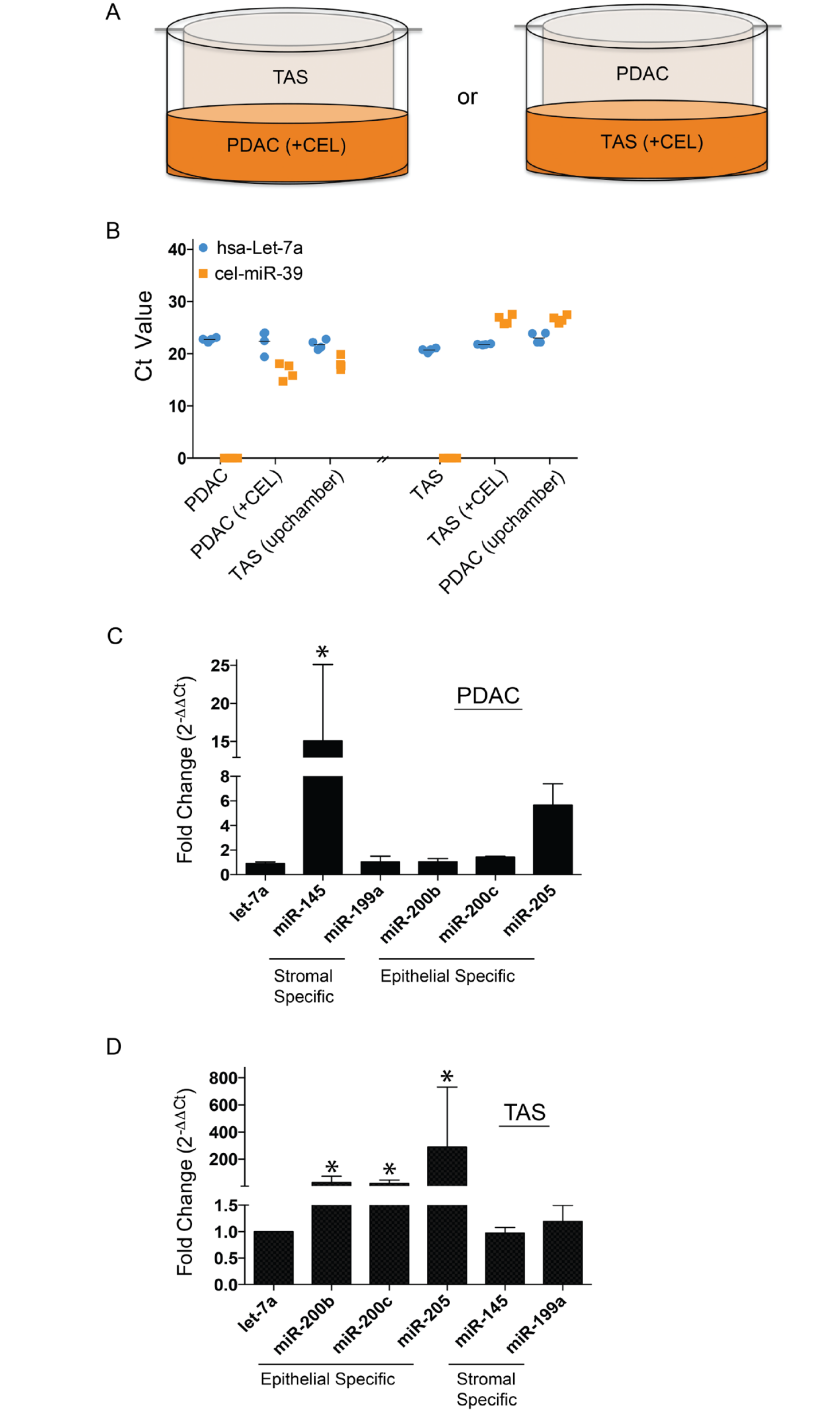
miRNA exchanges between PDAC and TAS cells are cell-cell contact independent (**A**) Design of Transwell^®^ co-culture with CEL transfected donor cells (+CEL) in the bottom chamber and recipient cells placed in the top chamber. (**B**) qPCR determination of cel-miR-39-3p expression level in both CEL transfected donor cells (+CEL) and Transwell^®^ co-cultured recipient cells (upchamber). Each point represents the Ct value of duplicate PCR reactions from two individual transfections and Transwell^®^ co-culture experiments. +CEL: cel-miR-39-3p transfected cells. (**C**) PDAC cells Transwell^®^ co-cultured with TAS cells and (**D**) TAS cells Transwell^®^ co-cultured with PDAC cells: qPCR determination of specific miRNAs. Bars represent means ± SDs of fold changes based on ΔΔCt calculation using hsa-let-7a-5p expression for normalization. ^*^p < 0.05.

We next asked whether cell-cell contact is necessary for this observed exchange of miRNA between cell types. To test this, we used polyester membrane cell culture inserts (Transwell^™^). CEL-transfected donor cells were plated in the bottom chamber and recipient cells in the top chamber (Figure 2A). qPCR again detected cel-miR-39-3p expression in both donor cells (PDAC or TAS cells with CEL-transfection) collected from bottom chambers as well as recipient cells (TAS or PDAC cells respectively) collected from top chambers (Figure 2B), indicating that the miRNA exchange between neighboring PDAC and TAS cells is not cell-cell contact dependent.

We previously reported the observation that cell-type-specific miRNA levels are increased in neighboring counterpart cells following monolayer co-culture [8] thus, we set to confirm that these changes in native miRNA expression concentrations also occur independent of cell-cell contact. As shown in Figure 2C and 2D, expression of TAS-specific miR-145 was detected by qPCR in PDAC cells co-cultured in inserts with TAS cells, and vice versa, epithelium-specific miR-205 and miR-200b/-200c were also detected in TAS cells. These data suggested that PDAC or TAS cells release miRNAs into culture media, and these miRNAs penetrate into recipient cells via a mechanism that is independent of cell-cell contact.

### miRNAs are selectively enriched as EVs cargo

**Figure 3:**
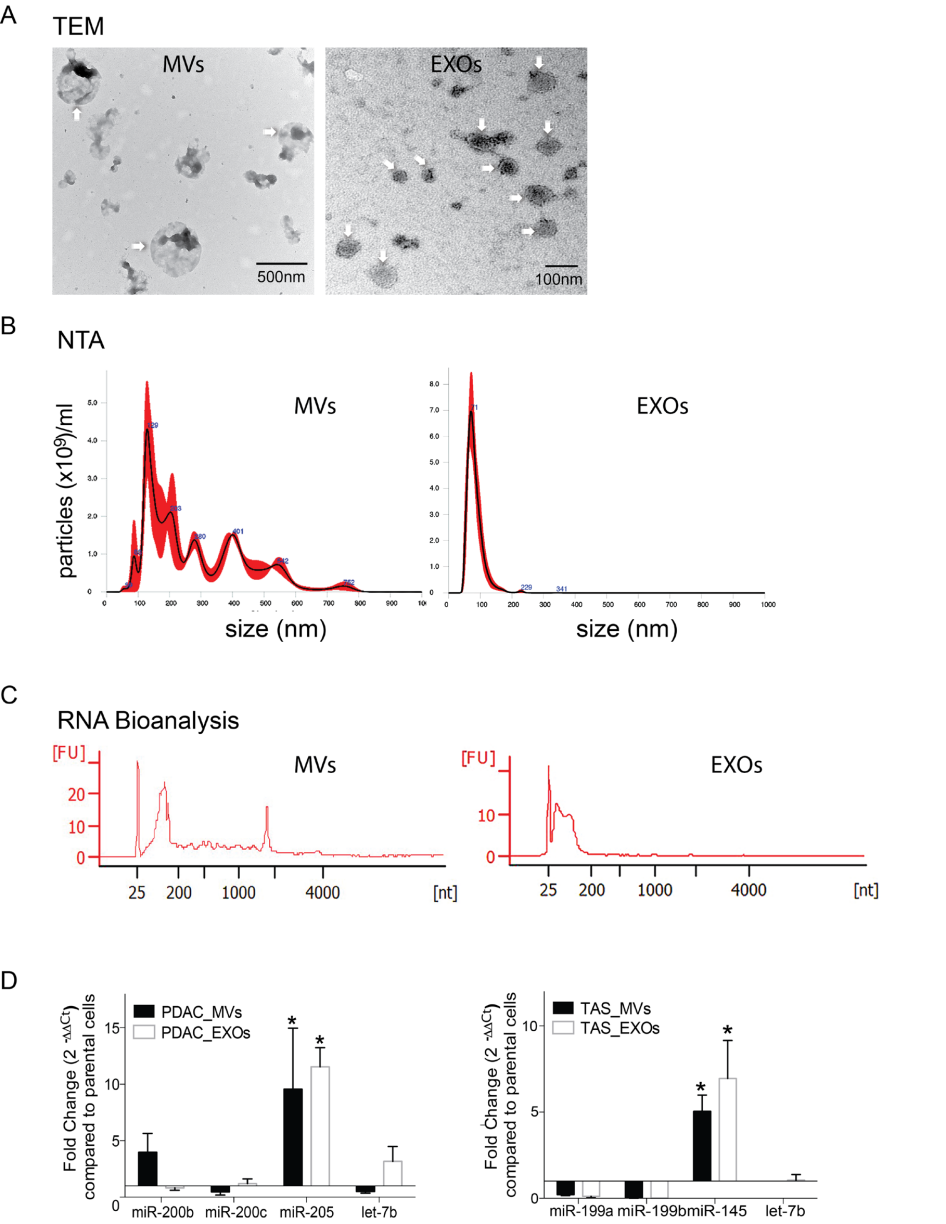
miRNA concentrations selectively enriched in EVs (**A**) Representative transmission electronic microscopic (TEM) images of MVs (digital magnification ×25000) and EXOs (digital magnification ×100000). Arrowheads indicate the membrane-bound nanoparticles of microvesicles (MVs) and exosomes (EXOs) as indicated. (**B**) Nanoparticle tracking analysis (NTA) measurements for isolated MVs and EXOs. The NTA graphs illustrate size distribution (0–1000 nm) v.s. concentration (particle number × 10^9^/ml). (**C**) Representative electropherogram of Agilent RNA Bioanalyzer 6000 pico analysis for the determination of concentration and size range of the isolated RNA fragments from MVs and EXOs. (**D**) qPCR detection of selective packing of specific miRNAs in EVs isolated from conditioned media from PDAC and TAS cell culture. Fold changes were normalized with hsa-let-7a-5p expression and calculated using ΔΔCt method comparing with expression levels in parental cells. EVs isolated from PDAC cells are collectively termed as PDAC-MVs or PDAC-EXOs, whereas EVs from TAS cells are collectively termed TAS-MVs or TAS-EXOs. Bars represent means ± SD. ^*^p < 0.05.

EVs could contain miRNAs [15]. Thus, we hypothesized that EVs are responsible for the miRNA exchanges in our PDAC/TAS cell co-culture model. Microvesicles (MVs) and exosomes (EXOs) are the two major subpopulations of EVs released by most types of cells. Differential ultra-centrifugation preparation was employed to isolate and segregate MVs and EXOs from serum-free, conditioned media from PDAC or TAS cell cultures [16, 17]. Segregation by size characterization of MVs and EXOs was confirmed by transmission electron microscopy (TEM) and NanoSight™ nanoparticle tracking analysis (NTA) measurement (Figure 3A and 3B). The average size for MVs and EXOs measured by NTA ranges from 60–600 nm and 30–160 nm respectively. In line with other reports [16, 18], Agilent RNA Bioanalyzer™ data revealed that MVs contained a mixed population of small RNAs with low-level expression of ribosomal RNA subunits, while EXOs predominantly contained RNAs smaller than 200 nucleotides (Figure 3C).

Next, total RNA was isolated from both purified MVs and EXOs and assayed for miRNA expression levels using qPCR. Indeed, qPCR detected the presence of PDAC signature miRNAs miR-200b, miR-200c and miR-205 in both MVs and EXOs released from PDAC cell lines. It was also noted that miR-205 expression levels were significantly elevated in MVs and EXOs as compared to the parental PDAC cells (difference = 8.53 ± 2.23, *p <* 0.001 for PDAC-MVs; and 10.55 ± 2.00, *p <* 0.001 for PDAC-EXOs). Likewise, TAS miRNA signature miR-145 and miR-199 family members miR-199a/199b were present in MVs and EXOs derived from TAS cells, and miR-145 concentrations were augmented in comparison to parental cell levels (difference = 4.04 ± 0.58, *p <* 0.001 for PDAC-MVs; and 5.95 ± 0.75, *p <* 0.001 for PDAC-EXOs) (Figure 3D). These data suggest selective packaging of miRNA cargos into EVs, resulting in the enrichment of certain miRNAs within EVs.

### Donor cell miRNAs contained in EVs are taken up by recipient cells

**Figure 4:**
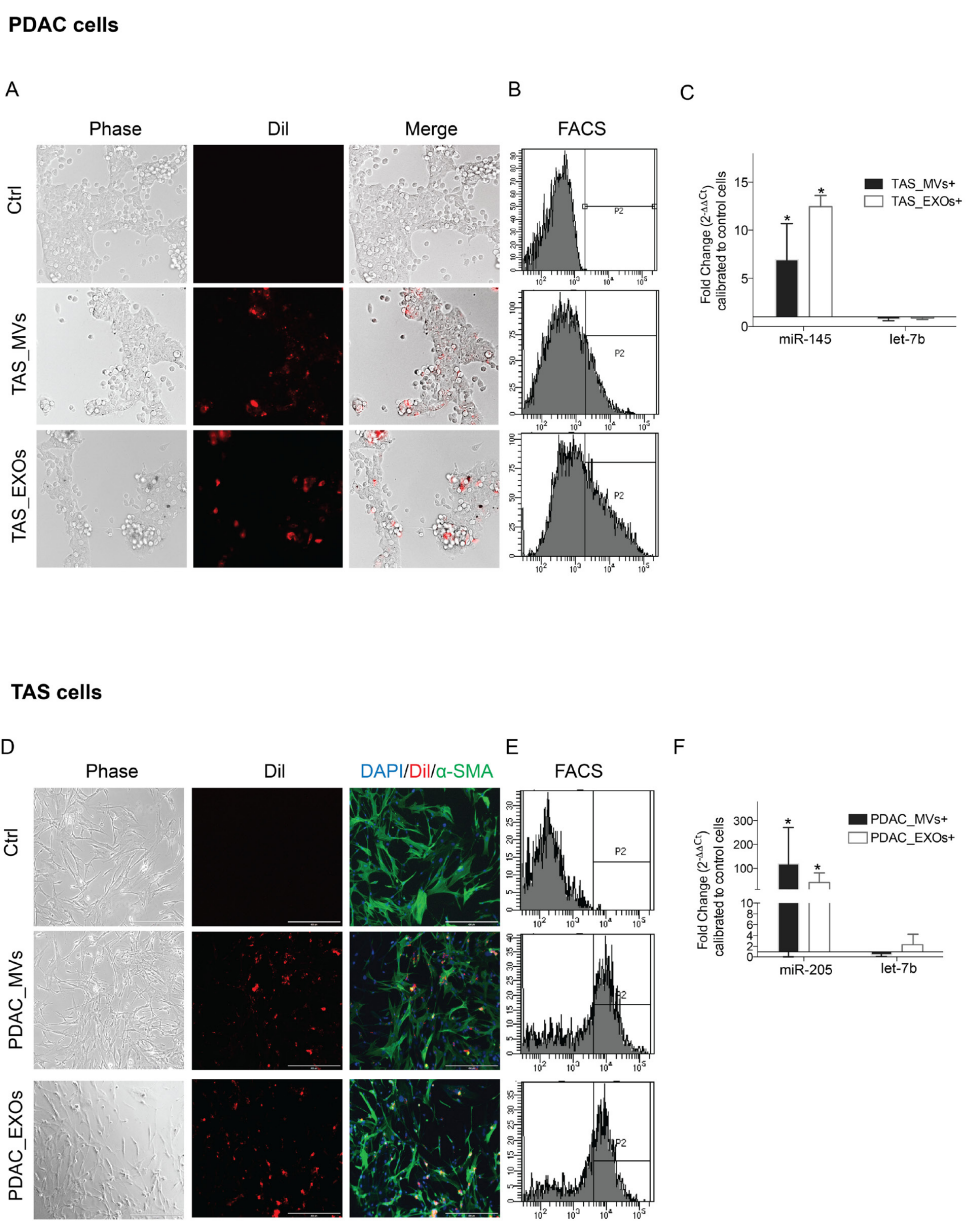
Recipient cells uptake EVs from conditioned medium Phase contrast and fluorescent microscopic images demonstrating engulfed DiI-labeled TAS-MVs and TAS-EXOs in recipient PDAC cells (**A**), and conversely engulfed DiI-labeled PDAC-MVs and PDAC-EXOs in TAS cells (**D**). Images were taken 12–16 hrs after EVs were added to the culture medium. Images were captured using Evos digital microscopy with 10× objective. (**B**) and (**E**) Flow cytometricc analysis confirmed a population of red fluorescence positive cells present in EV-fed groups. (**C**) and (**F**) qPCR detection of increased stromal specific miR-145 concentrations in PDAC cells fed with TAS-MVs and TAS-EXOs; and increased epithelial miR-205 concentrations in TAS cells fed with PDAC-MVs and PDAC-EXOs. Fold changes were normalized with hsa-let-7a-5p expression and calculated using ΔΔCt method comparing with expression levels in control cells. Bars represent means ± SD. ^*^p < 0.05.

To demonstrate that recipient cells internalize EVs released from donor cells in the tumor microenvironment, recipient cells (PDAC or TAS cells) were incubated with DiI-labeled MVs or DiI-labeled EXOs prepared from donor cells (TAS or PDAC cells, respectively) for 12–16 hours (Figure 4A and 4D). To further support the internalization of EVs rather than adherence to the surface of the target cell, recipient cells were trypsinized for extended times (5 min longer for PDAC cells and 10 min longer for TAS cells), pipetted, and vortexed vigorously to dissociate any potential membrane binding of EVs, and this was followed by flow cytometric determination of red fluorescent cells further gated by cell size and granularity (Figure 4B and 4E).

With this confirmation of recipient cells internalizing EVs from donor cells, we next aimed to provide further evidence that EVs transfer miRNAs into recipient cells. As expected, miR-145, a TAS signature miRNA, was present in PDAC cells fed with TAS-MVs or TAS-EXOs (Figure 4C). Vice versa, concentrations of the epithelial signature miRNA miR-205 were significantly increased in TAS cells fed with PDAC-MVs and PDAC-EXOs (Figure 4F). Taken together, these data strongly support the theory that both PDAC and TAS cells are capable of transferring specific miRNAs into counterpart cells via EV carriers.

### miR-145 inhibits PDAC cell viability and induces cell death

**Figure 5:**
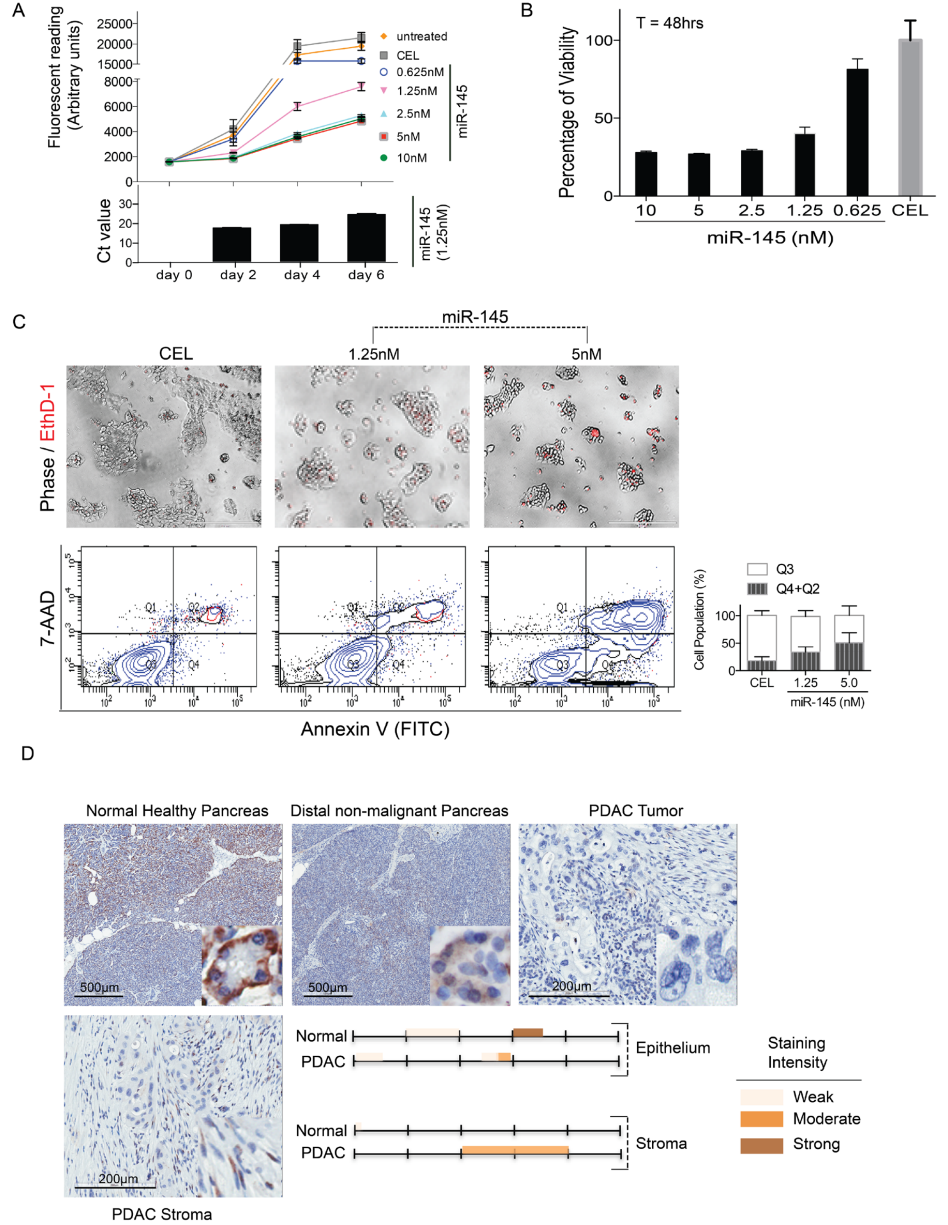
miR-145 inhibits PDAC cell proliferation and induces cell death For cell proliferation measurement (**A**), PDAC cells were plated in 96-well with 2000 cells/well and maintained in standard growing conditions for 6 days following CEL (5 nM) or miR-145-5p mimic transfections (0.625–10 nM). miR-145 levels during the 6-days of transfection period were representatively demonstrated using 1.25 nM transfection concentrations (bottom panel). For cell viability assessments (**B**), PDAC cells (20,000 cells/well) were plated and AlamarBlue^®^ assay was performed 48 hrs post transfection. Fluorometric readings were taken after 3 hrs of incubation with the reagents. (**C**) Cell death was observed under phase contrast and fluorescent microscopy using EthD-1 to label the dead cells (top panel). Cellular apoptosis was further analyzed using Annexin V/7-AAD flow cytometry analysis. Bars represent mean ± SD of three independent experiments (bottom panel). (**D**) ISH staining for miR-145-5p expression in human normal pancreas and PDAC tissue specimen. Staining intensity was color-indicated as shown.

We have previously demonstrated that exogenous over-expression of miRNAs miR-200b/-200c and miR-205 functionally altered TAS cells biologic activities by inducing cytokine production and inhibiting cell migration [8]. We expected that TAS-derived miRNAs would impact PDAC cell biology and thus investigated the impact of miR-145-5p released from TAS cells on PDAC cells. Post-transfection levels of miR-145 did persist at least 6 days (Figure 5A bottom). Compared to controls (untreated cells and cel-miR-39-3p transfected cells), PDAC cells with exogenous over-expression of human hsa-miR-145-5p mimic (final concentration of 0.625–10 nM) showed a dose-dependent decrease in cell proliferation rates and impaired cell viability (Figure 5A and 5B). miR-145-induced PDAC cell death was quantified using annexin V/7-AAD bivariate flow cytometric analysis and EthD-1 staining of necrotic cells (Figure 5C). The percentage of viable cells (Q3) decreased by 17.45 ± 6.37% (*p <* 0.05) and 32.80 ± 6.40% (*p <* 0.001) with miR-145 exogenous expression at concentration of 1.25 nM and 5 nM respectively; apoptotic/necrotic cells (Q2 + Q4) increased 15.68 ± 6.37% (*p <* 0.05) and 33.00 ± 6.38% (*p <* 0.001) with miR-145 exogenous expression at concentration of 1.25nM and 5nM respectively.

Evidence that miR-145 is a tumor suppressor in PDAC has been forwarded by others, but predominantly represents observations in animal models [19, 20]. We thus extended our study to explore miR-145-5p expression in human tissue samples of normal healthy pancreas, non-malignant pancreas remote from the malignancy, and PDAC. As shown in Figure 5D, in normal human pancreatic tissue, there is patchy cytoplasmic staining of the acinar cells with a moderate to strong intensity (approximately 60 to 70%). Weak, nuclear staining is appreciated in a minority of pancreatic ducts, with reactivity noted in approximately 10–20% of those structures. Conversely, ductal-like cancer cells in cases of PDAC lack any staining for miR-145-5p. However, significant, moderate intensity of miR-145-5p staining was observed in adjacent TAS in these samples of PDAC (Figure 5D). Taken together, our data suggest that miR-145-5p exerts an anti-tumor role in PDAC.

### TAS-derived EXOs induce PDAC cell death

**Figure 6:**
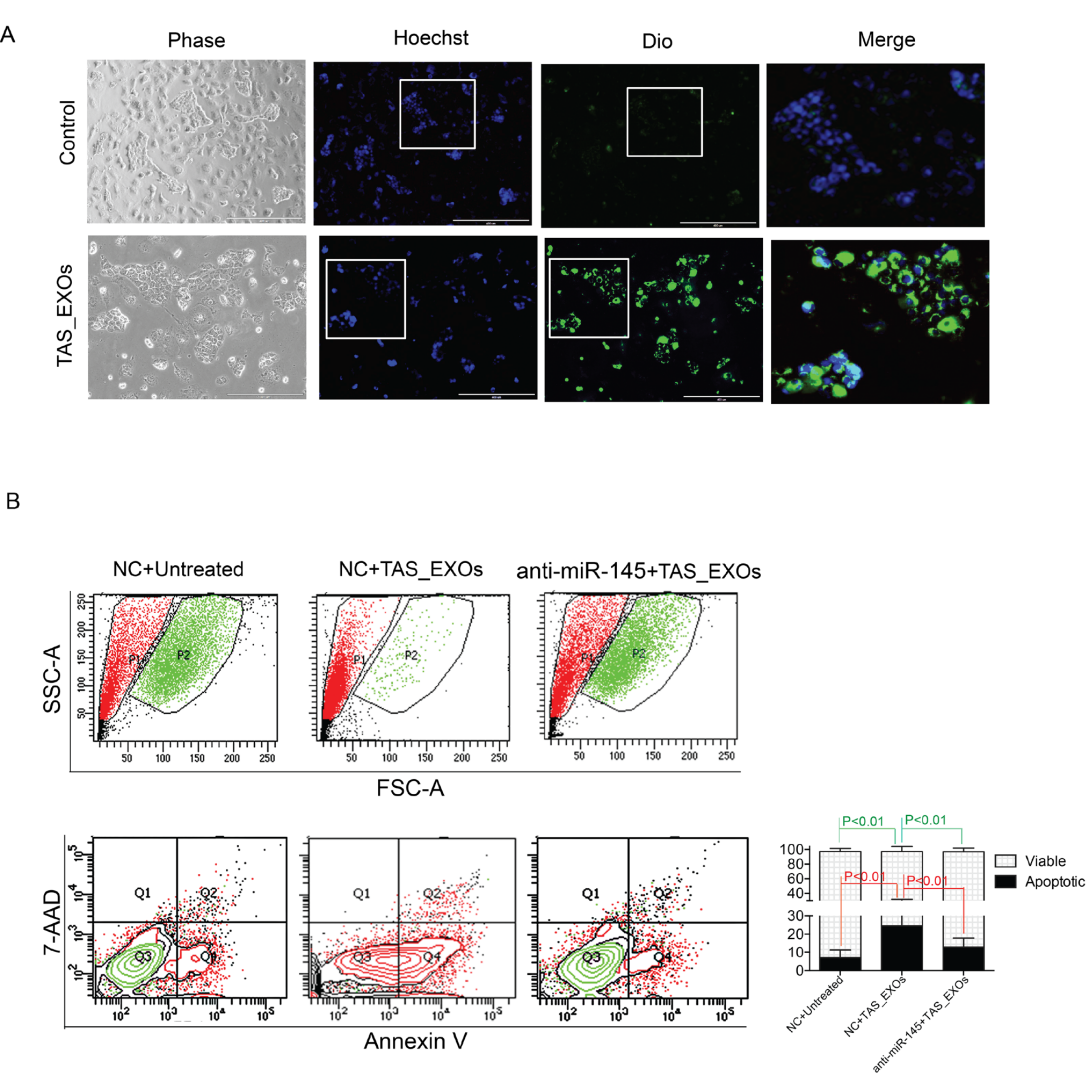
Exosomes derived from TAS cells induced PDAC cell death is mitigated by a miR-145 inhibitor (**A**) Representative phase/fluorescent microscopic images of PDAC cells in supplement with or without TAS-EXOs. Objective 10×. Blue: Hoechst 33442, green: DiO. Highlighted areas (white squares) are shown in merged images. (**B**) Flow cytometric assessment of undergoing apoptosis on the granularity (SSC) and size (FSC) distribution (top panel), as well as analysis for Annexin V/7-AAD staining (bottom panel). Results from one representative experiment of 10,000 events (cells) are shown. Bars represent cell populations of Q1 and Q3 from two independent experiments.

Based on the above data, we asked the question whether EVs derived from TAS cells could similarly impact PDAC cells via miR-145 cargo. To test this hypothesis, DiO-labeled TAS-MVs and TAS-EXOs were added to the PDAC culture medium. PDAC cells engulfing TAS-EXOs were recorded with fluorescent, microscopic imaging (Figure 6A). PDAC cells were fed with TAS-EXOs two-days in a row and cell growth/cell death was quantified on the third day. Cells in control group (with sham TAS-EXOs feeding) showed a healthy appearance and expansion to near confluence without observable green fluorescence in the cells. PDAC cells fed TAS-EXOs were confirmed to have ingested the EXOs (Figure 6A). In contrast to controls, these cells did not progress to confluence and appeared stressed with vacuolization and chromatin condensation (Figure 6A). As shown in Figure 4B, flow cytometric analysis confirmed the induction of cell apoptosis following treatment with TAS-EXOs. Cells undergoing apoptosis change with respect to granularity and cell volume, measurements readily identified by flow cytometric analysis. We observed a sub-population of cells (P2) with larger cell volume that consisted of over 98% of viable cells (Q3) that was reduced to less than 5% of viable cells in PDAC cells treated with TAS-EXOs (Figure 6B, top panel). TAS-EXOs-induced apoptosis was further confirmed by Annexin V analysis (Figure 6B, bottom panel). Perhaps most important, a miR-145 inhibitor was able to prevent/rescue this apoptotic population. In contrast, we could not make the same conclusion regarding the impact of TAS-MVs on PDAC cells. These data demonstrated that highly concentrated EXOs derived from TAS cells may have the capacity to confer a tumor suppressive role on adjacent PDAC cells via the delivery of miRNAs such as miR-145.

## DISCUSSION

We demonstrate that TAS-derived EVs are engulfed by adjacent PDAC cells and convey a tumor suppressive message via cargo miRNAs. These findings offer a potential mechanism to explain why stroma depletion strategies led to the acceleration of PDAC progression in murine models. Further studies to explore the translation of these findings to therapeutic strategies for PDAC are needed.

Cell-to-cell communication is essential to physiologic and pathologic processes, including cancer biology. Recent studies have led to the realization that in addition to long-known means of communication between cells, membrane-bound vesicles derived from cells convey other molecular cargos that function as signals to adjacent and/or remote targets [21]. This mechanism of cell-cell communication in the biology of cancer is an area of active investigation, but for solid organ malignancies, most studies to date focus on cancer-cell-derived EVs as mediators of the biology of non-cancer cells such as endothelial cells, immune cells and fibroblasts [22–24]. Conversely, very little is known regarding how such mechanisms might facilitate the influence of the stroma on the fate of cancer cells.

Pivotal findings of stroma-derived EVs signaling to neighbor cancer cells have been recently documented. For example, Luga and colleagues demonstrated that breast cancer associated fibroblasts produced EXOs that promote breast cancer cell motility and invasion through Wnt-planar cell polarity signaling pathways [25]; Zhao *et al.* reported that EXOs released from cancer-associated fibroblasts from prostate cancer patients contain intact metabolites (amino acid, lipids, etc) that can be utilized by cancer cells, thus promoting tumor growth under nutrient deprivation or stressed conditions [26]. Most relevant to the present work, EXOs derived from immortalized pancreatic stellate cells or cancer-associated fibroblasts have been reported to promote survival and proliferation of pancreatic cancer cells [27, 28].

In line with these studies, our data support the notion that TAS cells engage in communication with PDAC cells using EVs as vehicles to deliver molecular messages that influence tumor biology. Yet, our data differs from these prior reports with the novel finding that primary cultures of TAS produce EVs that may confer properties of tumor suppression. Our work supports and builds upon the putative, tumor suppressor role of miR-145 that has been experimentally supported by numerous authors [29–31]. Specific to PDAC, miR-145 expression in PDAC cells suppresses growth and invasion, and increases sensitivity to gemcitabine chemotherapy [20, 32]. Our data reproduce and expand upon these prior findings demonstrating over-expression of miR-145 in PDAC cells results in cell death. The mechanism of miR-145 in apoptosis is complicated and mediated through multiple cellular pathways, including caspase-dependent and -independent cell death [29, 33]. Oncogenic KRAS was reported to transcriptionally repress miR-143/145 cluster in a RREB1-dependent manner [19]. However, we further expand upon this finding by identifying that the source of miR-145 in the tumor microenvironment is actually from TAS cells that is transmitted to the PDAC cells via EVs. Of note, the other two stroma-specific miRNAs we previously identified, the miR-199 family members (miR-199a/-199b), have also been identified as tumor suppressive miRNAs [34, 35]. Favorably and in further support of our findings, other authors also reported pancreatic cancer-associated stromal EXOs enrich tumor suppressive miRNAs including miR-146a, miR-451a, and miR-630, etc. [36–41]. Considering that one microRNA can target hundreds of genes, we suspect that the mechanism of miR-145 regulation may be cell type specific. Future study to examine the role of miR-145 in large cohorts of patient’s tissues, especially its correlation with prognosis of the disease, will further define the potential therapeutical value of this miRNA.

One limitation of this study is of using monolayer cultures, further confirmation of these findings could be achieved by employing 3D culture of organoids to better resemble the tissues of origin. Another limitation is we utilized highly-expressing miR-145 TAS cells in this study. How stroma cell heterogeity may impact our findings is intriguing and challenging to determine. There are multiple types of fibroblasts in the pancreatic cancer stroma [7, 42, 43], i.e. Öhlund *et al.* identified two subtypes of PSC-derived CAFs in PDAC with distinct transcriptional profiles [43], further challenging the traditional view of uniformly protumorigenic role of tumor stroma. More work is needed to uncover the potential of divergent roles of various stromal cell populations in PDAC.

This work also builds upon our understanding of the exchange of membrane-bound molecular messages in the microenvironment by specifically exploring differential roles for EXOs and MVs (see [44] for review). EXOs are smaller vesicles (transitionally considered 30–120 nm) that originate from endocytic compartments within the cell, while MVs represent larger sized particles (transitionally considered 200–1000 nm) that are formed by budding directly from the plasma membrane. Differential ultracentrifugation does not provide pure populations of these differing EVs. Thus, we added additional ultrafiltration with (0.1 μm PVDF membranes) to minimize the MVs contamination in EXOs subpopulation preparation. Nonetheless, our technique is unlikely to provide a pure population of EXOs to completely distinguish the biological properties of TAS-derived MVs compared to EXOs and this is an obvious limitation to our conclusions here. Harastzi *et al.* reported the differential proteomic and lipidomic profiles for MVs and EXOs [45], and this work inspired us to test whether the signature miRNAs are differentially packed into MVs and EXOs. Although both TAS-MVs and TAS-EXOs contain miR-145 (detected by qPCR), TAS-MV failed to show the consistent and significant induction of apoptosis of PDAC cells. Whether this is due to the different EV content, the quantity of EV particles added to the culture medium, or differences in particle uptake by target cells is difficult to discern from our experiments, but certainly appears worthy of further investigation. On another note, the tumor microenvironment is more complex *in vivo,* involving other non-tumoral cells such as immune cell populations which could also influence the fate of cancer cells and our current experiments do not recapitulate this complexity such as how EVs may impact immunity. For example, the use of native EVs from mesenchymal stem cells and antigen presenting immune cells has attracted attention as a novel cell-free approach for cell therapy of various diseases [46].

In conclusion, our data suggest TAS cells orchestrate an intricate crosstalk with PDAC cells utilizing EVs to instigate anti-tumor signaling, thus providing insight into a potential mechanism to explain the proven, protective role of TAS [4–6]. How this biology of the anti-tumor property of TAS cells could be harnessed for therapeutic strategies remains undiscovered.

## MATERIALS AND METHODS

### Cell culture, monolayer co-culture, and Transwell co-culture

Immortalized PDAC cell lines (L3.6pl, BxPC3, MiaPaCa-2 and Panc-1) and primary, human xenograft-isolated PDAC cell lines (PC1, PC2, LM1 and LM2) as well as primary human TAS cell lines (TAS31, TAS32, TAS43, TAS58, TAS92) were cultured in DMEM-F12 supplemented with 10% FBS as previously described [47, 48]. All TAS cells used in this study are primary with between passage 4–6 (doubling time between 10–14 days) [47]. For the monolayer co-culture, PDAC cell lines and TAS cells were deposited on the culture surface at a ratio of 1:10 for immortal PDAC cell lines and 1:5 for xenograft primary PDAC cell lines respectively. The ratio between PC cells and TAS cells in monolayer co-culture was defined in order to achieve a histologic ratio at 48-hours of co-culture that closely mimics that of PDAC patient tissues [47]. For Transwell co-culture experiments, recipient cells were placed at the top chamber with donor cells at the bottom chamber.

### Fluorescence-activated cell sorting (FACS)

In order to separate monolayer co-cultured PDAC cells and TAS cells, all cells were detached from the culture surface with 0.05% trypsin, and then labeled with PE conjugated anti-ESA (epithelial surface antigen) antibody [8, 47]. To assure the purity of the ESA^–^ cell population, only cell lines with greater than 98% ESA^+^ cells were used in monolayer co-culture. ESA^+^ (representing PDAC cells) and ESA^–^ (representing TAS cells) were separately collected. Sorted cells were then subjected to RNA extraction.

### RNA extraction and quantitative real-time PCR (qPCR)

As previously describe [8], cultured cells were lysed in Trizol (Invitrogen, Carlsbad, CA), followed by phenol-chloroform phase separation of nucleic acids. RNA Agilent pico Bioanalyzer (Agilent Technologies, Waldbronn, Germany) was performed to assess RNA/small RNA concentrations. Reverse transcription was performed from the RNA samples using Universal cDNA synthesis kit (Exiqon, Denmark). miRNAs were amplified with predesigned primer sets [8] and the miRCURY LNA^™^ Universal RT microRNA PCR system (Exiqon), and amplifications were carried out on a Mx3005p thermocycler (Strategene, La Jolla, CA). hsa-let-7a, a miRNA, consistently and abundantly expressed in both stromal and cancer cells, was utilized as an internal control and for normalization. Fold differences between groups were calculated using ΔΔC_T_ methods.

### miRNA mimics and inhibitors transfection

PDAC cells were plated in 6-well plates (2.5 × 10^5^ cells/well), 12-well plates (1.5 × 10^5^ cells/well), or 96-well plates (5 × 10^4^ cells/well) the day before transfection. Cells were transfected with synthetic miRNA hsa-miR-145-5p mimics or miRCURY LNA hsa-miR-145-5p inhibitors (Exiqon, Vedbaek, Denmark) using RNAiMAX transfection reagent (Thermo Scientific, Waltham, MA). Synthetic miRNA mimic from *c. Elegans* (Cel-miR-39-3p, CEL) and inhibitor control (NC A) served as controls.

### Cell proliferation, cell viability and cell death assessment

Cell proliferation and cell viability was assessed using alamarBlue^®^ assay (Thermo Scientific, Waltham, MA). Fluorescence intensity was measured after 2 hrs of incubation with 10 μl reagent at 37°C using a Clariostar plate reader (BMG Labtech, Cary, NC). Cell apoptosis and necrosis was determined with Annexin V/7-AAD (BD Biosciences, San Jose, CA) flow cytometery analysis. EthD-1 (ethidium homodimer-1, Sigma, St. Louis, MO) staining was visualized under fluorescence microscopy (Evos^®^ FL Imaging Systems).

### Preparation of microvesicles (MVs) and exosomes (EXOs)

In order to achieve large-scale EV production, media from six of T150 flasks of cultured cells were collected. The two subpopulations of EVs were prepared using differential centrifugation modified based upon protocols of serial centrifugation. Briefly, serum-free conditioned culture media were centrifuged at 2000 g for 30 min to clear dead cells and debris. Supernatants were then subjected to centrifugation at 20,000 g for 30 min to pellet MVs. Secondary supernatants were filtered through 0.1 μm membrane followed by ultracentrifugation at 100,000 g for 120 min to pellet the EXOs. Transmission electronic microscopy (TEM) was performed at the Electron Microscopy Core of University of Florida on a Hitachi 7600 transmission electron microscope (Hitachi High-Technologies America, Schaumburg, IL) equipped with a MacroFire^®^ monochrome progressive scan CCD camera (Optronics, Goleta, CA). Particles size and concentration was analyzed using NanoSight LM10 with Nanoparticle tracking analysis (NTA) software (Malvern, UK). All samples were pre-diluted with PBS to the desired concentration range of 1–9 × 10^9^ as recommended for NTA measurements.

### Assessment of *ex vivo* miR-145 expression

miR-145 expression in FFPE samples were quantified by *in situ* hybridization (ISH) performed by BioGenex Laboratories Inc. (Fremont, CA) with miR-145 ISH probes (100 nM) and Super Sensitive One-step Polymer-HRP ISH detection kit (HM145, DF400, BioGenex laboratories, Fremont, CA). Immuno-reactivity was visualized with DAB with hematoxylin counter staining. FDA scoring staining system was applied with strong staining given a score of 3 (+); moderate staining a score of 2 (+); and weak staining a score of 1 (+); and tissues lacking staining entirely were scored 0 (−). The final report was generated based on two independent scores by our GI pathology specialists.

### Statistical analysis

All statistical analysis was conducted using Prism v6 software. Group comparison was analyzed with two-way repeated measures ANOVA. Statistically significance was defined as a probability of *p <* 0.05.

## Abbreviations

PDAC: pancreatic ductal adenocarcinoma
TME: tumor microenvironment
EV: extracellular vesicles
MV: microvesicles
EXO: exosomes
TAS: tumor-associated stroma
